# Anticancer activity of *Pseudomonas aeruginosa* derived peptide with iRGD in colon cancer therapy

**DOI:** 10.22038/IJBMS.2023.68331.14913

**Published:** 2023

**Authors:** Atieh Yaghoubi, Aref Movaqar, Fereshteh Asgharzadeh, Mohammad Derakhshan, Kiarash Ghazvini, Seyed Mahdi Hasanian, Amir Avan, Asma Mostafapour, Majid Khazaei, Saman Soleimanpour

**Affiliations:** 1 Department of Microbiology and Virology, Faculty of Medicine, Mashhad University of Medical Sciences, Mashhad, Iran; 2 Student Research Committee, Faculty of Medicine, Mashhad University of Medical Sciences, Mashhad, Iran; 3 Department of Physiology, Faculty of Medicine, Mashhad University of Medical Sciences, Mashhad, Iran; 4 Department of Medical Biochemistry, Faculty of Medicine, Mashhad University of Medical, Sciences, Mashhad, Iran; 5 Department of Medical Genetics and Molecular Medicine, Faculty of Medicine, Mashhad University of Medical Sciences, Mashhad, Iran; 6 Department of Biology, Mashhad Branch, Islamic Azad University, Mashhad, Iran

**Keywords:** Azurin-p28, Bacterial peptide, Colon cancer, Irgd, Pseudomonas aeruginosa

## Abstract

**Objective(s):**

Colon cancer is well-known as a life-threatening disease. Since the current treatment modalities for this type of cancer are powerful yet face some limitations, finding novel treatments is required to achieve better outcomes with fewer side effects. Here we investigated the therapeutic potential of Azurin-p28 alone or along with iRGD (Ac-CRGDKGPDC-amide) as a tumor-penetrating peptide and 5-fluorouracil (5-FU) for colon cancer.

**Materials and Methods:**

Inhibitory effect of p28 with or without iRGD/5-FU was studied in CT26 and HT29, as well as the xenograft animal model of cancer. The effect of p28 alone or along with iRGD/5-FU on cell migration, apoptotic activity, and cell cycle of the cell lines was assessed. Level of the BAX and BCL2 genes, tumor suppressor genes [(p53 and collagen type-Iα1 (COL1A1), collagen type-Iα2 (COL1A2)] were assessed by quantitative RT-PCR.

**Results:**

These findings show that using p28 with or without iRGD and 5-FU raised the level of p53 and BAX but decreased BCL2, compared with control and 5-FU groups in tissues of the tumor, which result in raising the apoptosis.

**Conclusion:**

It seems that p28 may be used as a new therapeutic approach in colon cancer therapy that can enhance the anti-tumor effect of 5-FU.

## Introduction

More than 1 million new cases of colon cancer were expected to be detected in 2020, making it the second most diagnosed cancer among women and the third most prevalent among men (1). Traditional therapies for patients suffering from colon cancer, including surgery, chemotherapy, and targeted therapies, have faced limitations in the treatment of this type of cancer (2). Lack of specific toxicity toward tumor cells is among the disadvantages of conventional anti-cancer therapies. According to the reports, conventional therapies using chemotherapeutic agents can’t be effective in lesions that occur in the advanced stages of colon cancer and trans-coelomic spread across the peritoneal cavity (3, 4). Chemotherapy and targeted therapy drugs are unable to reach these lesions, resulting in treatment failure and a general survival rate of only a few months (5, 6). Colon cancer surgery also has several disadvantages, especially in the elderly population, raising the risk of post-operative side effects and perioperative mortality (7, 8). Thus, new therapeutics with fewer side effects are desperately needed to get over these restrictions.

Using bacteria as therapeutic agents for cancer has currently aroused attention in medical and pharmaceutical studies (9-11). William Coley used the *Serratia marcescens* and *Streptococcus pyogenes* for the first time in 1909, in treating 1000 patients with unresectable cancers for the first time. This mixture was known as Coley’s toxins (12). Meanwhile, *Bacillus Calmette–Guérin* (BCG), i.e., was used in the 1970s as therapy for those who live with non-muscle-invasive bladder cancer (NMIBC), which was approved by the FDA. (13, 14). Among the different metabolites of bacteria, peptides with bacterial origin have drawn a lot of interest because of their distinctive qualities, such as their small size, easily adjustable features, and quick and typically straightforward synthesis (15). Furthermore, bacterial peptides demonstrate high specificity in inhibiting tumor cell growth, and also they are quite potent in penetrating the cell membranes (16). Evidence suggests that bacterial peptides have minimum drug-drug interaction (17, 18). Azurin-p28 (Compare Azurin-p28 vs. p28) is one of the main bacterial peptides in cancer therapy produced by *Pseudomonas aeruginosa*  (19, 20). This peptide has 28 amino acid lengths derived from Azurin (21). P28 has received more attention recently due to its high anticancer potential for several cancers (19, 22). Azurin-p28 has a preferentially penetrating impact on the tumor cells. It can also inhibit tumor cell proliferation as well as cause tumor cell shrinkage and death through (i) binding to domain DNA (DBD) of p53, (ii) anti-generation potential, as well as (iii) inducing apoptosis (23). In addition, this peptide can inhibit angiogenesis in cancer cells (23). iRGD (CRGDKGPDC) has a 9 amino-acid cyclic length that is a tumor-homing peptide for cancer therapy (24). iRGD can penetrate the cancerous cells through integrins called αVβ3 and αVβ5, and then a cleavage by protease leads to activation of the peptide c-terminal CendR motif (R/KXXR/K) (25). For the first time, we used p28 in co-administration with iRGD as a tumor-penetrating peptide. We herewith investigated the anticancer effect of Azurin-p28 with or without the tumor-homing peptide iRGD and 5-FU on *in vitro* and *in vivo *models of colon cancer.

## Materials and Methods

***Peptide synthesis and purification***

ProteoGenix Inc. produced the p28 anticancer peptide (LSTAADMQGVVTDGMASGLDKDYLKPDD, 2914 Da) (20) and the iRGD homing peptide (Ac-CRGDKGPDC-amide), a disulfide-based cyclic peptide, with >95% purity and mass balance. After the linear chain amino acid was finished, this process was oxidized on solid-phase resin by thallium trifluoroacetate. Mass spectrometry and amino acid analysis were used to further analyze the isolated peptides.

***Cell culture***

Cell lines for the human colon cancer HT29, murine colorectal carcinoma CT26, and normal fibroblast L929 were obtained from the Pasture institute of Tehran (Iran). In Roswell Park Memorial Institute (RPMI) 1640 (Gibco Life Technologies, Thermo Fisher Scientific, MA, USA) with fetal bovine serum (FBS) (10% v/v), penicillin (100 U/ml), and streptomycin (100 U/ml) in a humid environment at 37 ^°^C with 5% CO_2_, all three cell lines were thereafter cultivated.

***Cytotoxicity assessment using MTT assay***

All three cancer cell lines were cultured in a culture plate with 96 wells for 24 hr. Cells were subsequently subjected to five different treatments p28, p28+iRGD, p28+5-FU, p28+iRGD+iRGD, iRGD, and 5-fluorouracil for 24 hr at varying doses (Ebewe Pharma, Austria). The cells were then treated with MTT (0.05 mg/ml, per well) for 4 hr at 37 ^°^C. DMSO (100 l per well) after the supernatant was eliminated. Determination was done by using a microplate reader (TECAN NanoQuant Infinite M200 Microplate Reader, USA) with absorbance at 540 nm. Three separate MTT tests were conducted.

***Apoptosis analysis by flow*** ***cytometry***

For our proposal, we utilized an annexin V apoptosis detection kit (MabTag GmbH, Germany). CT26 and HT29 cells in the logarithmic growth phase (2-3 105 cells/well) were planted onto 6-well plates. After a 24 hr culture period at 37 ^°^C and 5% CO_2_, a fresh medium containing p28, p28+iRGD, p28+5-FU, p28+5-FU+iRGD, iRGD, and 5-FU was used. After that, the cells were washed with PBS and centrifuged at 400 g for five minutes. Annexin V-FITC/PI double labeling method was used for the apoptosis assay. After centrifuging the cells, 90 ml of binding buffer was added, and 5 ml of PI and 5 ml of annexin V-FITC were added to stain the cells (with incubation for 20 min. in the dark). Tagged cells were then analyzed using a FACSCalibur flow cytometer, and the labeled cells were gathered for examination.

***Cell cycle analysis***

The following 24 hr were spent treating CT26 and HT29 cells in 6-well plates with p28, p28+iRGD, p28+5-FU, p28+5-FU+iRGD, iRGD, and 5-FU. Then, RNase A was added to the mixture then incubated at 37 ^°^C for 30 min. After the initial incubation period, the cells were treated with propidium iodide (PI, 50 µg/ml), and incubated at 24 ^°^C for 20 min. After the labeled cells were gathered for flow cytometric analysis with the FACSCalibur flow cytometer, the cell cycle was then studied using FlowJo V10-CL software.

***Migration assay***

After being sown in the 12-well plates, the cells were expanded until they were 70% confluent. For the migration assay, by using a p200 pipette tip, the cells were scratched, and the free unattached cells were then removed using PBS. A 10× objective microscope was used to measure the cell-free area (ZEISS Microscopy, Germany). To assess the migration of transfected cells, untransfected cells as a control group were used. ANOVA test was used to determine whether there were any significant differences between the transfected and non-transfected cells after each assay was carried out in triplicate.

***In vivo antitumor efficacy***

Thirty-six inbred BALB/c mice, with an age of 7 weeks, were obtained from the Pasteur Institute in Tehran, Iran, and kept there under guidelines endorsed by the Institute of Animal Ethics Committee (IR.MUMS.MEDICAL.REC.1398.899) (22±2 ^°^C, 54±2% humidity, and a 12 hr light/dark cycle). Mashhad University of Medical Sciences Ethical Committee approved the Care and Use of Laboratory Animals Guidelines, which were followed by all protocols. The mice’s left flank was subcutaneously injected with CT26 cells (2×10^6^).

**Figure 1:**
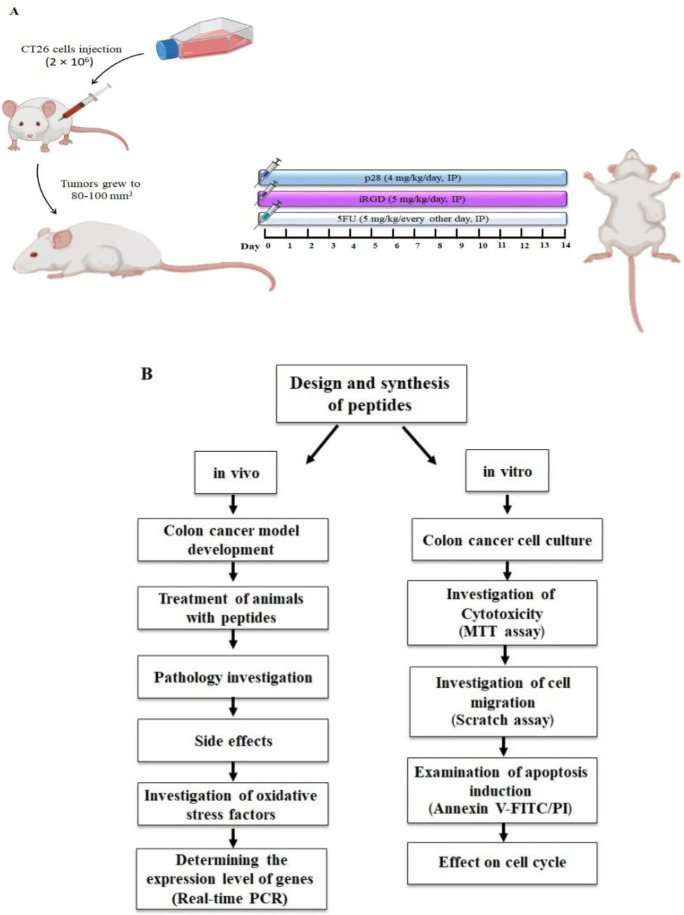
A: Schematic representation of the experimental methodology and mouse colon model; B: Workflow

The xenograft mice were divided into seven groups (n=6 in each group) after the tumors reached a size of 80-100 mm^3^: (i) control (tumor not receiving treatment); (ii) 5-FU (administered with a 5 mg/kg intraperitoneal (IP) injection every other day). The following substances are listed in order of increasing potency: (iii) p28 (4 mg/kg/day, IP); (iv) iRGD (5 mg/kg/day, IP); (v) p28+iRGD (4 mg/kg/day and 5 mg/kg/day IP, respectively); (vi) p28+5-FU (4 mg/kg/day and 5 mg/kg/every other day IP, respectively) (26-28) (Figure 1). Every other day, the tumor size was measured with a digital caliper using the formula: Tumor volume=(tumor length)×(tumor width)2/ 2. A full dose of Ketamine-Xylazine (87 mg/kg-13 mg/ kg) was utilized to quickly create a deep state of unconsciousness, and the cervical dislocation technique was subsequently employed to sacrifice the mice (29). The mice were dissected after the experiment. The mice were dissected after the experiment. Major organs (heart, liver, lungs, and kidneys) and tumor tissues were removed for histological analysis.

***Histological evaluation***

Major organs like the heart, liver, lungs, and kidneys were removed and cut into 5 μm slices. We used Hematoxylin-Eosin (H&E) and Masson’s trichrome stains for histological evaluation. The percentages of necrosis and fibrosis were estimated using ImageJ software (NIH, Bethesda, MD, USA).

***Oxidative stress factors measurement***

By homogenizing tumor tissues, 3 indicators of oxidative/antioxidative stress were determined, including malondialdehyde (MDA), total thiol groups (SH), superoxide dismutase (SOD), and catalase (CAT) activity. MDA was measured to investigate the antioxidant activity of p28 alone or recombined with iRGD or/and 5-FU. A 10% homogenate of tissue of each tumor was blended with 1 ml of trichloroacetic acid, thiobarbituric acid, and hydrochloric acid solution (30). The SH groups were recognized by the DTNB (Di-Tio nitro benzoic acid) reagent. Reacting the SH groups with DTNB reagent produced a yellow color complex (31). To ascertain the auto-oxidation of pyrogallol and suppress the conversion of MTT to formazan, SOD activity was assessed (32). Catalase enzyme activity was examined by the Aebi method. The process is based on H_2_O_2_ being hydrolyzed in a buffer of phosphate (pH 7.0). Under normal circumstances, the activity of CAT was created by the conversion of H_2_O_2_ to H_2_O and O_2_ in 1 min (33, 34).

***RNA isolation and real-time PCR***

Favorgen Biotech’s FavorPrepTM Tissue Total RNA Mini Kit (Taiwan) was used in this study. A kit of cDNA synthesis, available from Yekta Tajhiz, was also used to create the cDNA (Tehran, Iran). Roche Diagnostics (Mannheim, Germany) light cycler real-time PCR was used for the gene expression study. The reaction mixture for the PCR technique contained the following components per 25:400 nM per primer, 1.25 U of Taq polymerase, 5.5 mM MgCl_2_, 200 M deoxynucleotide triphosphate (dNTP), and 5 l of template DNA make up the 1X buffer mixture. On a thermal cycler, the PCR was cycled 40 times at 95 ^°^C for 30 sec, 60 ^°^C for 30 sec, and 72 ^°^C for 30 sec, with the final extension taking place at 72 ^°^C for 7 min. The initial denaturation was done at 95 ^°^C for 5 min. Bringing the gene expression levels to a standard reference gene (GAPDH). To ascertain the relative changes in gene expression, findings were examined by 2^-Delta Delta C(T)^.

***Statistical analysis***

The results were examined by means and standard deviations. An LSD *post hoc* test was used after a one-way ANOVA test to compare different groups. The data were analyzed using SPSS Ver. 20, a statistical tool (IBM, Chicago, USA). A *P*-value lower than 0.05 was used to define statistical significance

## Results

***In vitro activity***

**Figure 2:**
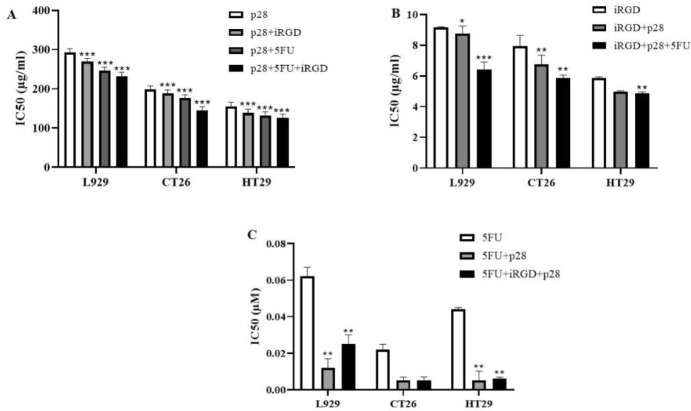
IC_50_ values for the peptide-treated L929, CT26, and HT29 cells

Antitumor effects were examined, the viability of all three cell lines were determined by MTT assay, and findings were analyzed by CalcuSyn software (Ver. 2.0). Different p28 concentrations were applied to cells, either with or without a 6 µg/ml tumor-targeting peptide and 3.84×10^-5 ^M 5-FU for 24 hr. The viability of the cancer cell lines drastically declined. Even while a high dosage of iRGD alone produced cytotoxic effects on the cancer cells, the combination of iRGD and p28 resulted in a greater inhibition. The concentration of peptides increased while the HT29 cell line’s cell viability drastically reduced. The CT26 cell line, in contrast, saw a sluggish drop, indicating that the HT29 cell line was more responsive to the combination therapy. Furthermore, exposure to 119.68 µg/ml p28 for 24 hr, with or without the addition of 6 µg/ml of iRGD and 3.84×10^-5 ^M 5-FU, had no effect on the proliferation (5%) of normal fibroblasts (Figure 2).

***Apoptosis induction activity***

**Figure 3:**
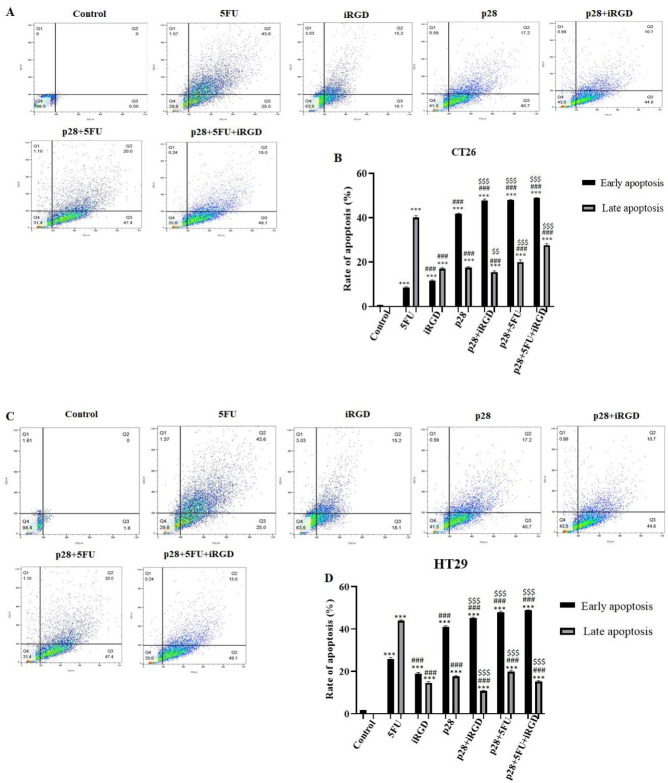
Ability of peptides to cause apoptosis in cancer cell lines

Apoptotic activity was calculated by flow cytometry after both cell lines were grown with 119.68 μg/ml p28 alone or in combination with iRGD and 5-FU. FlowJo-V10 software was used to examine the findings of the flow cytometry. Thus, a crucial metric for determining the anticancer effects of these two peptides *in vitro* is the cancer cells’ apoptotic rate following the co-administration of this peptide and p28. Figure 3 displays the findings, which revealed that co-administration of p28 with iRGD and 5-FU could significantly increase the apoptosis rate of CT26 and HT29 cells compared with a single therapy. 

Additionally, treatment with p28 with or without iRGD and 5-FU significantly enhanced the level of BAX while decreasing the level of BCL2 in the tumor tissue, which increases the ratio of apoptosis (*P*<0.01 and *P*<0.001, respectively). Co-administration of p28 in combination with iRGD and 5-FU dramatically up-regulated the expression of the BAX gene while also drastically down-regulating the expression of BCL2 (*P*<0.001) (Figure 3E).

***Role of p28 in the cell cycle regulation***

**Figure 4:**
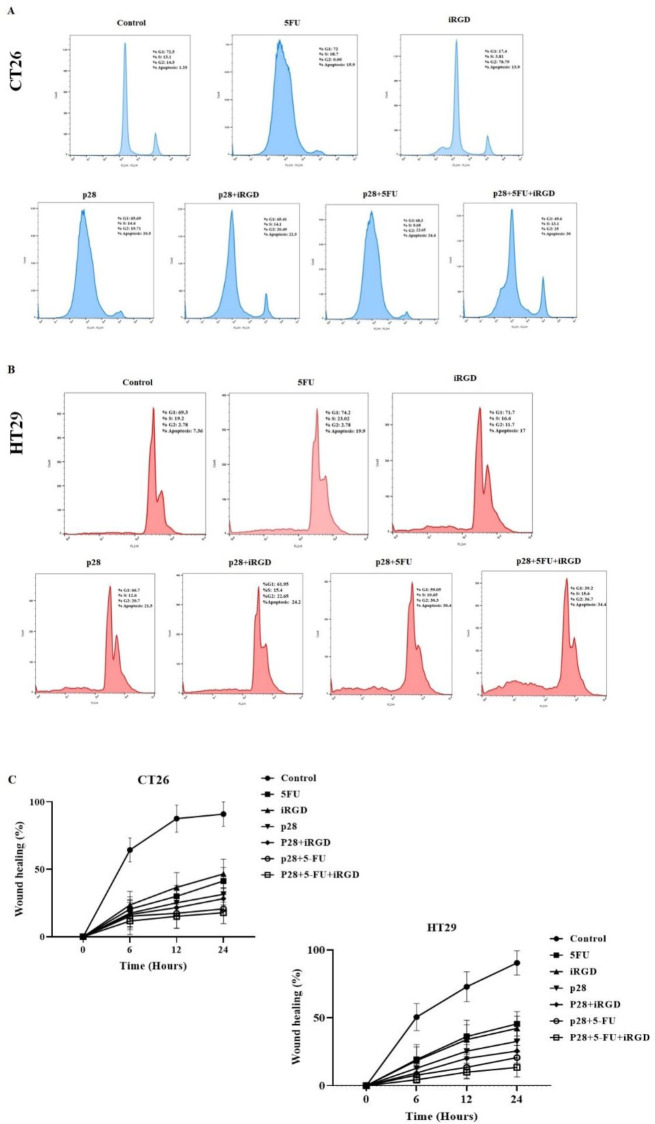
Shows how colon cancer cell lines’ cell cycles are affected by p28

We assessed the activity of p28 alone or with 5-FU and iRGD on the growth cycle of cells by flow cytometry. The results of the flow cytometry were examined using FlowJo-V10 software. 119.68 µg/ml p28 was added to CT26 and HT29 cells for 24 hr along with either 5-FU or iRGD. Similar to the results for apoptosis, co-administration of p28 with iRGD and 5-FU led to noticeably more CT26 and HT29 cells in sub-G1 than when p28 was given alone (20.5% and 21.5%, respectively). Additionally, analyses of the cell cycle in the CT26 and HT29 cell lines demonstrate that p28 with 5-FU and iRGD raised the cell population at the G2-M phase (25% and 36.7%, respectively) after 24 hr in comparison with the cells treated with p28 alone (19.71% and 20.7%, respectively). Additionally, p53 levels in the tumor tissues were markedly elevated with p28 alone or with 5-FU and iRGD (*P*<0.001). In the xenograft tumor tissues, the therapy of p28 with 5-FU and iRGD dramatically raised the amount of p53 expression (*P*<0.001). (Figures 3 and 4).

***Migration assay***

**Figure 5:**
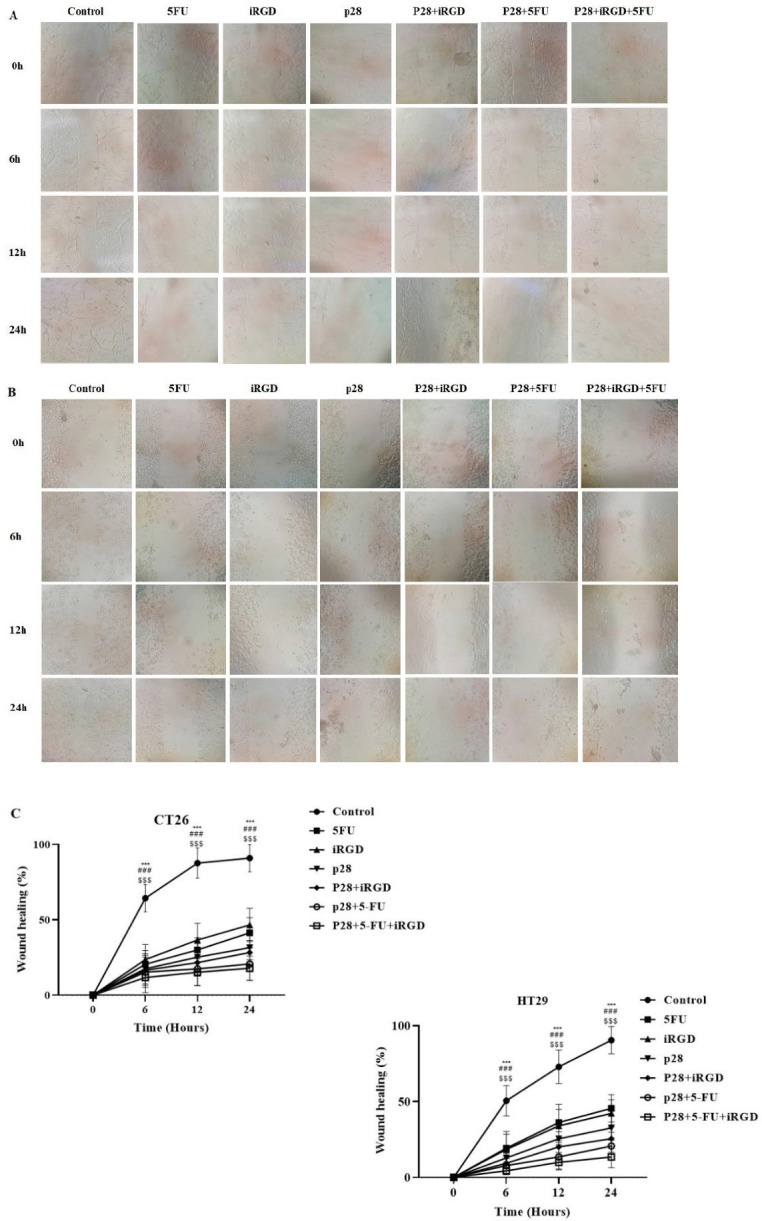
Shows how p28 prevents colon cancer cells from migrating and encroaching

We used the wound healing method to determine the impact of p28 alone or in conjunction with iRGD/5-FU on the control of cell migration. NIH, Bethesda, Maryland, developed ImageJ software to calculate the rate of wound healing (40). 119.68 µg/ml p28 alone or with 5-FU and iRGD can significantly prevent cell migration over time, according to cell migration studies of the CT26 and HT29 cell lines. Additionally, the results of both cell lines show that, compared with cells exposed to p28 alone after 24 hr, combined therapy of p28 with iRGD and 5-FU can considerably block cell migration [(prevention rate of CT26: 17.91% and 31.58%, respectively); (prevention rate of HT29: 13.45% and 32.64%, respectively)] (Figure 5).

***In vivo antitumor efficacy***

**Figure 6:**
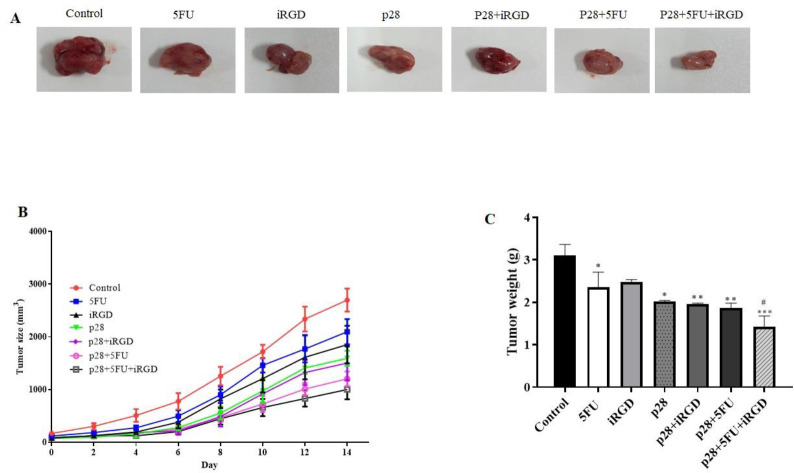
shows how p28, either alone or in combination with iRGD and 5-FU, inhibits the development of CT26 cells in xenograft nude mice

**Figure 7:**
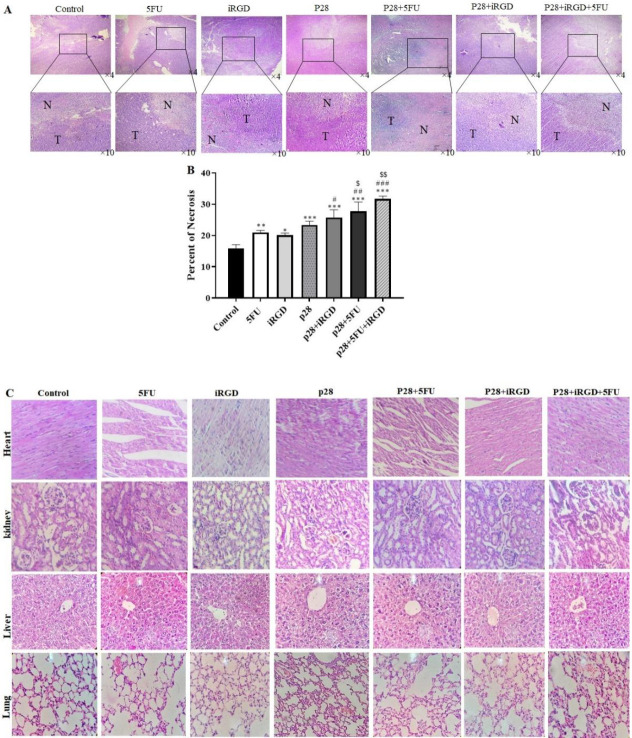
Shows microscopic findings from the xenograft mouse model showing necrosis and its impact on organs

**Figure 8:**
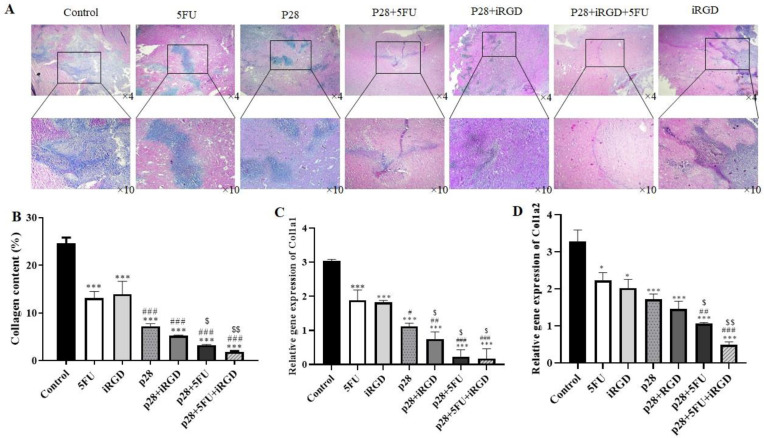
Trichrome staining shows the presence of fibrotic tissue and an increase of collagen in tumor tissue

These results demonstrated that p28 therapy alone or in combination with iRGD or/and 5-FU significantly reduced tumor size and weight. Furthermore, these results showed that the combined treatment of p28 with iRGD and 5-FU may significantly reduce the tumor size and weight (*P*<0.001 and *P*<0.05, respectively) (Figure 6). We performed H&E staining on the tumor tissues, and the results showed that, when p28 with iRGD and 5-FU was used in combination therapy, it markedly raised the necrotic area in the tumor compared with the control group and p28 and 5-FU alone. Additionally, the combination of p28 and 5-FU therapy enhanced the tumor (Figure 7). Trichrome staining results also revealed that treatment with p28 alone or in combination with iRGD or/and 5-FU markedly decrease the fibrotic area and collagen formation in the tumor. However, the collagen accumulation, as well as fibrotic area in the animals, were considerably reduced when p28 was also administered along with iRGD and 5-FU (*P<*0.01). Additionally, p28 and 5-FU co-administration markedly reduce collagen accumulation as well as fibrotic area when compared with p28 or 5-FU alone (*P*<0.05 and *P*<0.001, respectively) (Figure 8). When 5-FU was treated alone or in combination with iRGD or/and 5-FU, CoL1a1 and CoL1a2 gene expression was significantly reduced in the tumor tissues (*P*<0.001). Additionally, p28 co-administration with iRGD and 5-FU, or therapy with p28 and 5-FU, dramatically decreased the expression of the CoL1a1 and CoL1a2 genes in the tumor tissues when compared with the mice receiving solo therapy with p28 or 5-FU (*P*<0.05 and *P*<0.001, respectively). The H&E results of the major organs (heart, liver, lung, and kidneys) of each group were utilized to evaluate the potential toxicity and likely side effects of providing p28 alone or in combination with iRGD and 5-FU. No discernible changes were seen histologically (Figures 7 and 8).

***The impact of p28 on inflammation and the balance of oxidants and antioxidants***

**Figure 9:**
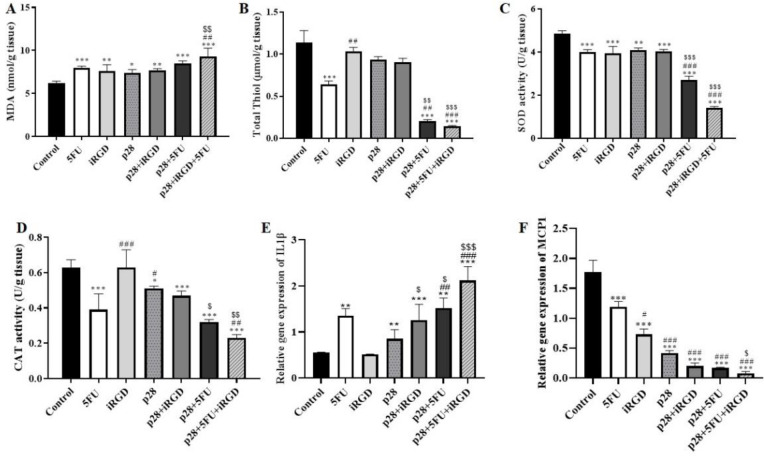
Shows the impact of p28 on indicators of oxidative, cytokines of inflammation, as well as genes that promote fibrosis

In tumor tissue homogenates, we evaluated the impact of p28 on the balance of oxidant as well as antioxidant markers. These findings showed that xenograft models receiving single therapy with either p28 or 5-FU, along with p28 with 5-FU and iRGD had dramatically reduced anti-oxidant indicators such as thiol, catalase, and SOD activity (*P*<0.001). While p28 co-administration with iRGD and 5-FU significantly elevated the malondialdehyde (MDA) level indicators of oxidative stress (*P*<0.001 and *P*<0.01 respectively), the xenograft models received sole therapy with either p28 or 5-FU (Figure 9). Moreover, we found that mono-therapy with p28 peptide can increase the pro-inflammatory cytokines. Furthermore, our results demonstrated the effect of p28 on xenograft models that received a single therapy with either p28 or 5-FU, considerably raising the inflammatory genes such as IL1β that were elevated in the treatment of p28 with 5-FU and iRGD (*P*<0.001). (Figure 9E). Additionally, treatment of p28 alone with iRGD or/and 5-FU dramatically decreases the pro-fibrotic gene MCP-1 (*P*<0.001) (Figure 9F). The recent findings demonstrated that the disruption of the balance of oxidant as well as antioxidant markers and elevation of the pro-inflammatory cytokine, which result in cancer cell death, were likely the mediators of the tumor-suppressive action of p28 on colon cancer models.

## Discussion

At the moment, bacterial peptides are gaining popularity as a cutting-edge method of treating cancer. p28 is well-known as a small peptide derived from Azurin which is a multitarget antitumor agent. Here, we examined the impact of p28 peptide anticancer therapy as monotherapy or combination therapy along with iRGD and 5-FU *in vitro* and animal models of colorectal cancer for the first time. We found that p28 with tumor-penetrating peptides of iRGD and 5-FU increased the apoptosis rate of CT26 and HT29 cells significantly. In addition, we found that co-administration of p28 with iRGD and 5-FU down-regulated BCL2 in the tumor tissue homogenates. BAX also known as a Bcl-2-like protein 4 has a role in cell death and apoptosis regulations (35). Additionally, proapoptotic genes like BAX lose their ability to act as tumor suppressors due to overexpression of BCL2. Therefore, suppressing or controlling BCL2 expression can enable tumor cells to resume their natural apoptotic process (36). With a very short half-life and functions like tumor suppression, p53 is involved in the control of the cell cycle. Ubiquitin-mediated pathways also control the basal concentration of p53 (37, 38). Breast, ovarian, hepatocellular, colon, and stomach cancers are only a few of the malignancies that have excessive levels of the p53-negative regulator COP1 (also known as COP1). Additionally, the p28-p53 complex promotes the transcription of genes known to interact with mitochondria, such as BAX and Noxa, which are pro-apoptotic (39-41). Cells were treated with p28 either alone or in combination with iRGD and 5-FU at the G2-M phase. In contrast to p28 or 5-FU monotherapy, the injection of p28 with 5-FU dramatically decreases fibrosis and collagen. According to earlier studies, treatment with the p28 peptide can diminish the phosphorylation of FAK and Akt, which inhibits the motility and migration of endothelial cells (23, 42, 43). The major structural element of the extracellular matrix and a prominent member of the collagen family is a type I collagen, which is well-known (44).

One chain of collagen type I 2 (COL1A2) and two chains of collagen type I 1 (COL1A1) make up this kind, which is a heterotrimer (45). The invasion and development of tumors are facilitated by type I collagen (46, 47). Numerous cancers, including colorectal cancer and medulloblastoma, have higher expression levels of COL1A1 and COL1A2. COL1A1 and COL1A2 expression changes are utilized to forecast prognosis in a number of cancer types (48-51).

According to earlier publications, our findings also showed that the combined therapy of p28 with iRGD and 5-FU dramatically decreased the CoL1a1 and CoL1a2 genes. Additionally, compared with a single therapy, p28 plus 5-FU combination therapy also dramatically reduces the expression of these two genes. Additionally, we discovered that the pro-inflammatory gene IL1 β was considerably up-regulated in the tumor tissue by the combination therapy of p28 with iRGD and 5-FU. It appears that p28 also has anticancer potential due to the disruption of the oxidant/antioxidant equilibrium brought on by the down-regulation of antioxidant indicators including thiol, catalase, and SOD, and the amplification of oxidative stress markers like MDA. All of these results suggested that p28 might be used as a colon cancer treatment medication. It can also enhance the effects of chemotherapy medications. 

One of the essential chemokines needed for all phases of tumor formation, including tumor initiation and metastasis, is MCP-1, which is well-known. Additionally, the tumor stage and grade in individuals with breast and bladder malignancies, as well as rises in colorectal cancer, are all positively connected with serum levels of MCP-1 (52-54). Our findings showed that treatment of p28 alone, with iRGD or/and 5-FU, down-regulated the level of MCP-1. Based on earlier studies, IL1β can trigger both Th1 and Th17 responses, which have an anti-tumorigenic effect (55, 56). When IL1β was injected into the xenograft models, the tumors shrank until they were large enough and the mice’s T cell counts were normal (57).

(A) After 24 hr, the MTT test was used to determine the IC50 of p28 alone or along with iRGD and 5-FU at the stated concentration. (B) After 24 hr, an MTT assay was used to determine the IC50 of iRGD alone or in combination with p28 and 5-FU at the stated concentration. (C) IC50 of 5-FU alone or in combination with iRGD and p28. (In comparison with cell lines treated with peptides or 5-FU alone, *P<0.05, **P<0.01, and ***P<0.001)

(A) For 24 hr, the 119.68 µg/ml p28 with or without 5-FU and iRGD was applied to CT26 cell lines. (B) Proportion of cells in late and early apoptosis in CT26 cells that had been exposed to the peptides shown in (A). (C) For a 24 hr period, HT29 cell lines were exposed to p28 alone or with iRGD and 5-FU. (D) The proportion of HT29 cells that underwent early and late apoptosis after being exposed to the peptides shown in (A). (E) BAX, BCL2, and p53 expression levels in the tumor tissue. (**P<0.01 and ***P<0.001 compared with the control group); (^##^*P<*0.01 and ^###^*P<*0.001 compared with the 5-FU group); and (^$$^*P<*0.01 and ^$$$^*P<*0.001 compared with the p28 group)

(A) CT26 cells as well as (B) HT29 cells received 119.68 μg/ml p28 for 24 hr, either with or without the addition of 5-FU and iRGD. To evaluate, the cell cycle utilizing PI flow cytometry was done. It shows the proportion of cells in G1, S, G2-M, and sub-G1 (apoptosis) phases

Effect of p28 inhibition of (A) CT26 and (B) HT29 following observation (×10 objectives; ZEISS Microscopy, Germany). (C) Proportion of both cancer cell lines that repair wounds

(A) Typical pictures of tumors from each therapy group. (B) The rate of tumor growth in each group was assessed throughout the trial. (C) Weight of each treatment group’s tumors. (#*P<*0.05 compare to 5-FU group); (*P<0.05; **P<0.01; and ***P<0.001 compare to control group)

(A) In each group, the tumor area is depicted by T, and the necrosis area is denoted by N. Each figure included a description of the magnification. (B) Necrosis. H&E staining was used to examine the morphological characteristics of the indicated organs in (C). Following therapy with p28 with or without iRGD or/and 5-FU, nude mice showed no discernible pathological alterations in the heart, liver, kidney, or lung. (*P<0.05, *P<*0.01, and *P<0.001 compared with the control group); (^$$^*P<*0.01 and ^$$$^*P<*0.001 compared with the p28 group); and (#*P<*0.05, ^##^*P<*0.01, and ^###^*P<*0.001 compared with the 5-FU group)

(A) Fibrosis is visible in the blue area. (B) Amount of collagen in a different group as a percentage. CoL1a1 and CoL1a2 gene expression levels in tumor tissue are shown in C and D. (*P<*0.05 and ^$$$^*P<*0.001 compared with the p28 group); (*P<*0.05 and ^$$$^*P<*0.001 compared with the 5-FU group); and (*P<0.05, **P<0.01, and ***P<0.001 compared with the control group)

Results of p28 alone or with 5-FU and iRGD: total serum thiol, MDA, SOD activity, and catalase activity (Figure 1A). Effects of p28 on levels of the IL1β and MCP-1, respectively, are shown in Figures (E and F). (*P<0.05, **P<0.01, and ***P<0.001 compared with the control group); (^$^*P<*0.05, ^$$^*P<*0.01, and ^$$$^*P<*0.001 compared with the p28 group); and (#*P<*0.05, ^##^*P<*0.01, and ^###^*P<*0.001 compared with the 5-FU group)

## Conclusion

We should also point out that a phase I clinical trial was conducted on young patients with recurrent or progressing central nervous system cancers to examine the safety, tolerability, pharmacokinetics, and activity of the p28 peptide. Due to the blood-brain barrier, which restricts a drug’s ability to reach the brain tumor, these sorts of cancers are frequently extremely aggressive and challenging to treat (58, 59). However, more investigations are needed to confirm the exact activity of p28 in inducing the suppression of colon tumors.

## Authors’ Contributions

AY and FA performed conceptualization, investigation, and writing; MK, KG, SMH, AA, AF, AM, and WCC reviewed and edited the manuscript. 

## Funding

This study was supported by Elite Researcher Grant Committee under award number [4000660] from the  National Institute for Medical Research Development (NIMAD), Tehran, Iran, and a grant from Iran National Science Foundation (98022042).

## Data availability statement

The datasets generated and/or analyzed during the current study are available from the corresponding author upon reasonable request.

## Conflicts of Interest

The authors have declared that have no conflicts of interest
