# Corrigendum: Anticancer activity of *Pseudomonas aeruginosa* derived peptide with iRGD in colon cancer therapy

**DOI:** 10.22038/ijbms.2025.25959

**Published:** 2025

The authors sincerely apologize for the error in the published original article entitled “Anticancer activity of Pseudomonas aeruginosa derived peptide with iRGD in colon cancer therapy”, which was published with incorrect pictures. Due to the authors’ request, the pictures below should replace the ones in Figure 5 (A, B, and C) and Figure 8 (A) in the original article. In the originally published version of the manuscript, Figure 5 (A and B) included data for the 6-hour (6 h) time point. The authors have removed the images related to this time point from the figures, as the slow proliferation cycle and migration rate of the cell lines (CT25 and HT29) studied is 24 hours, and biologically significant changes are not expected to occur within the first 6 hours. The authors also identified the growth margin line of the cells in the image. This revision aligns the presented data with the experimental rationale and does not affect the conclusions of the study. During the production process in Figure 8 (A), the incorrect figure for the 5FU group was inadvertently included in the final version. This oversight was identified post-publication, and the correct figures have now been verified and submitted to the journal for replacement.

**Figure:**
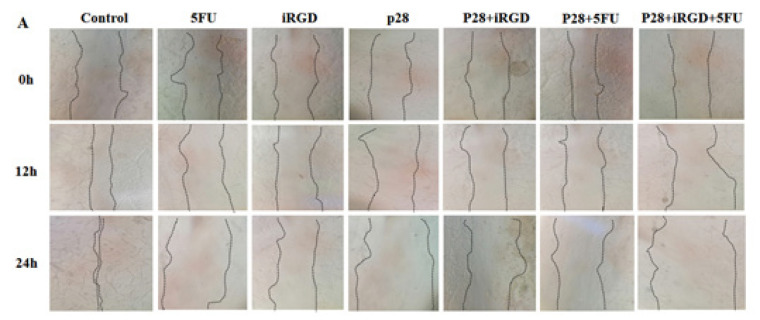
Figure 5A

**Figure:**
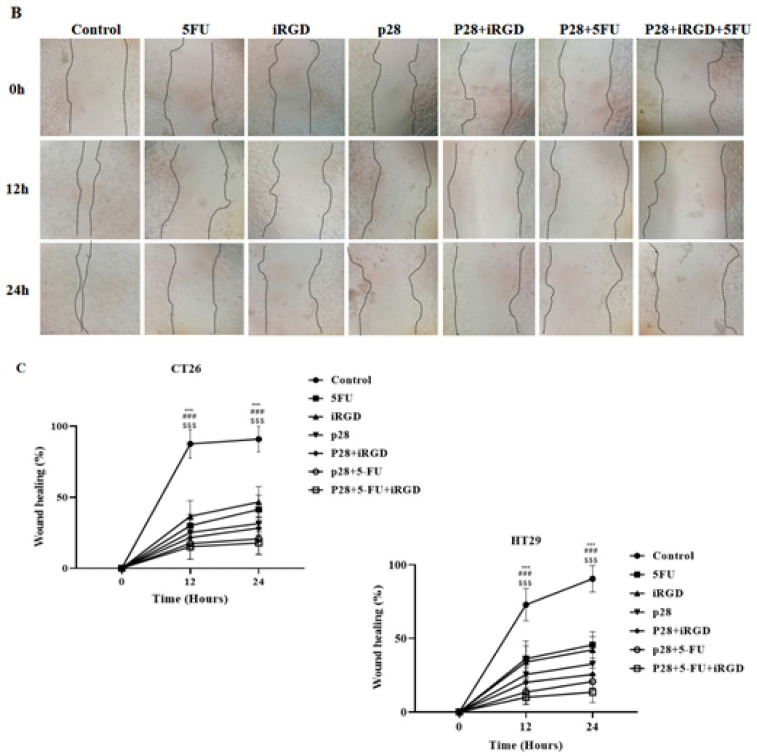
Figure 5B&C

**Figure:**
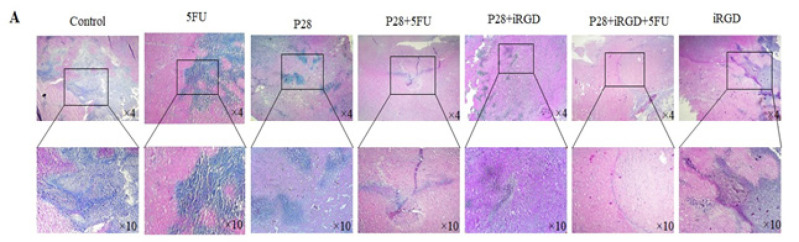
Figure 8A

In this article, we unintentionally failed to cite our previous publication: “Yaghoubi, A., Asgharzadeh, F., Movaqar, A. *et al. *Anticancer activity of Helicobacter pylori ribosomal protein (HPRP) with iRGD in treatment of colon cancer. J Cancer Res Clin Oncol 147, 2851–2865 (2021)”. Since both studies were conducted simultaneously, the Control group, 5-Fluorouracil (5- FU), and iRGD groups in both animal experiments and cell culture studies were shared between the two projects. This design was implemented to reduce animal use [based on the Animal Ethical Committee and 3Rs Principle (Reduction, Replacement, Refinement)] and ensure consistency in cellular experiments by maintaining identical culture conditions.



